# Porphyrin-based nanotechnology: a minimally invasive approach for drug delivery and cholesteatoma treatment

**DOI:** 10.3389/fsurg.2025.1594001

**Published:** 2025-09-03

**Authors:** Dina Ashraf Mahmoud, Lili Ding, Zannatul Ferdous, Zhifen Zhang, Juan Chen, Jennifer L. Spiegel, Edward Alexander Sykes, Robert Harrison, Gang Zheng, Trung Le

**Affiliations:** ^1^ Temerty Faculty of Medicine, University of Toronto, Toronto, ON, Canada; ^2^ Biological Sciences Platform, Hurvitz Brain Sciences Program, Sunnybrook Research Institute, Toronto, ON, Canada; ^3^ Faculty of Pharmacy, Alexandria University, Alexandria, Egypt; ^4^ Princess Margaret Cancer Centre, University Health Network, Toronto, ON, Canada; ^5^ Department of Otolaryngology—Head and Neck Surgery, University of Toronto, Toronto, ON, Canada; ^6^ Department of Otorhinolaryngology, Faculty of Medicine, Ludwig Maximilians University Munich, Munich, Germany; ^7^ Department of Surgery, Division of Otolaryngology Head and Neck Surgery, St. Joseph’s Health Centre, Unity Health Toronto, Toronto, ON, Canada; ^8^ Department of Otolaryngology—Head and Neck Surgery, University of Toronto, Toronto, ON, Canada; ^9^ Program in Neuroscience and Mental Health, Hospital for Sick Children, Toronto, ON, Canada; ^10^ Department of Pharmaceutical Sciences, University of Toronto, Toronto, ON, Canada; ^11^ Institute of Biomedical Engineering, University of Toronto, Toronto, ON, Canada; ^12^ Department of Medical Biophysics, University of Toronto, Toronto, ON, Canada; ^13^ Institute of Medical Science, Temerty Faculty of Medicine, University of Toronto, Toronto, ON, Canada

**Keywords:** porphysomes, photothermal therapy, cholesteatoma, inner ear drug delivery, hearing loss, regeneration, infection, nanoparticles

## Abstract

The treatment of inner and middle ear diseases remains a significant challenge, often requiring surgical intervention as the only option. In this study, we investigated porphysomes, self-assembled porphyrin-based nanoparticles, as a minimally invasive drug delivery platform for inner ear applications and as photothermal agents for cholesteatoma ablation. Three porphysome formulations were evaluated: parent porphysomes (PS), porphyrin-stabilized nanoemulsions (nPS), and EDTA-lipid incorporated porphysomes (ePS). Rats received intratympanic injections of each formulation, and fluorescence imaging performed 1 h postinjection demonstrated concentration-dependent inner ear penetration for all formulations. Notably, no signs of ototoxicity were observed based on histological and functional assessments of hearing and balance. Among ePS-treated rats, the 250 and 1,000 μM groups showed significantly higher inner ear fluorescence compared with the 50 μM group. Auditory brainstem response (ABR) and distortion product otoacoustic emission (DPOAE) tests were performed at baseline and 2 and 6 weeks postinjection. No significant threshold shifts were detected in PS and ePS groups compared with controls. In contrast, nPS-treated rats exhibited a significant ABR threshold elevation at 6 weeks (*p* < 0.02). At 6 weeks, minor yet statistically significant DPOAE threshold differences were observed at 16 kHz (PS group) and 32 kHz (all porphysome groups); however, all shifts remained below 0 dB, indicating no functional hearing loss. Vestibular assessments, including swim and beam tests, revealed no significant impairments. Upon laser activation, all porphysome formulations induced substantial temperature elevation (Δ*T* = 31 ± 3.76°C, *p* < 0.05) and histological burn effects in cholesteatoma tissues. These findings support the potential of porphysomes as a safe, minimally invasive drug delivery system and photothermal agent for the treatment of inner and middle ear disorders.

## 1 Introduction

Middle ear cholesteatoma is a destructive, non-neoplastic lesion driven by chronic inflammation, with an annual incidence of approximately 10 per 100,000. Characterized by keratinized squamous epithelium, cholesteatoma often invades the temporal bone, particularly the middle ear cleft, causing bone erosion and significant morbidity (1).

Histologically, cholesteatoma comprises three layers: the keratinized epithelial matrix, the connective tissue-rich perimatrix, and cystic contents composed of keratin lamellae. The perimatrix, containing collagen, fibrocytes, and inflammatory cells such as lymphocytes, plasma cells, and neutrophils, exhibits a thickness directly correlated with the degree of inflammation and disease severity (2, 3). This structural complexity contributes to the invasive behavior of cholesteatoma and its resistance to conservative treatments (4).

Surgery remains the primary treatment, but recurrence can reach 40%, and contralateral ear involvement is common in bilateral disease (1). This underscores the importance of follow-up and highlights the disease burden on healthcare systems (5). Laser therapy has become increasingly utilized in cholesteatoma. Among these, the potassium titanyl phosphate (KTP) laser operates at a wavelength of 532 nm and utilizes a semiflexible fiber-optic delivery system, allowing access to hard-to-reach areas within the middle ear. However, the non-specific nature of KTP laser treatment poses a risk of collateral thermal injury to critical structures such as the facial nerve, underscoring the need for more targeted therapies to safely manage cholesteatoma (6).

Cholesteatoma-induced chronic inflammation disrupts the round window membrane, enabling inflammatory mediators to infiltrate the inner ear and cause cochlear damage, including hair cell loss (7–9). Addressing both local tissue destruction and secondary inner ear inflammation requires a multifunctional approach. Porphysomes, as a versatile nanoplatform, combine photothermal cholesteatoma ablation with potential for targeted drug delivery to mitigate inner ear injury. This study evaluates their therapeutic efficacy, safety, and permeability to support future inner ear applications.

Porphysomes are ∼100 nm self-assembled nanoparticles composed of pyro-lipid units containing approximately 80,000 porphyrin molecules. They accumulate preferentially in solid tumors through the enhanced permeability and retention (EPR) effect and then are internalized by cancer cells and disassembled into pyro-lipid components. These nanoparticles have unique dual functionality; in their intact form, they enable photothermal therapy (PTT) by converting light to heat, while disassembly triggers fluorescence and reactive oxygen species (ROS) generation for photodynamic therapy (PDT). Their versatility, combined with compatibility for imaging modalities such as positron emission tomography and magnetic resonance imaging, makes porphysomes promising theranostic agents (10, 11). Porphysomes are the first single all-organic agent with the ability for concurrent phototherapy and drug delivery. At 671 nm, porphysomes exhibit super-quenching of porphyrin fluorescence, enhancing conversion of absorbed light to heat, which can ablate abnormal tissue such as cholesteatoma (12). Infrared thermography was the most practical monitoring method in our study. It has been clinically relevant for detecting abnormal increasing skin temperature, which stems from raised metabolic activity of an underlying tumor (13).

Advanced formulations from the prototype porphysomes, such as nanoemulsion porphysomes (nPS) and EDTA porphysomes (ePS), further expand their potential. nPS provides superior colloidal stability and targeted drug delivery capabilities. To add, the oil core enhances the efficient encapsulation of hydrophobic compounds such as paclitaxel, thus producing a multimodal nanoplatform for cancer imaging, phototherapy, and image-guided drug delivery which we aim to leverage for inner ear diseases (14).

The final porphyrin-based nanoparticle that was investigated in this study is called ePS. It is synthesized by incorporating aminopolycarboxylic acid-conjugated lipids, such as EDTA, into liposome-like porphyrin nanoparticles resulting in the formation of ePS. ePS showed significant enhancement of the cellular uptake by 25-fold as demonstrated in various studies (15–17). This enhancement was attributed to the ability of these lipids to fluidize the cell membrane in a detergent-like manner, instead of their metal chelation properties with EDTA or DTPA (18–20). ePS relies on this distinctive mechanism of cellular active uptake, achieving over 95% cell death through photodynamic therapy (PDT) when compared with PS alone which showed <5% cell death (11). EDTA has been shown to destabilize membranes and disturb tight junctions by intercalating with phospholipid molecules on the membrane and chelating Ca^2+^ ions (15, 19). These actions can aid in the uptake of drugs, and therefore in this study, ePS nanoparticles specifically are investigated for inner ear drug delivery.

Inner ear drug delivery faces several challenges (21). Physiological and anatomical characteristics of the inner ear, such as its limited blood supply and the presence of the blood-inner ear barrier, can hinder the access of systemic drugs to target cells within the cochlea. To add, short half-lives of systemically administered drugs, impaired parenteral absorption for high molecular weight substances, and considerable interindividual variations due to differences in body metabolism are further hurdles (22). The inner ear presents an ideal site for local drug delivery due to its isolation by the blood-inner ear barrier and the ability of perilymph and endolymph fluids to swiftly distribute liquids throughout the cochlea (23). Local delivery circumvents the blood-inner ear barrier, enabling drugs to reach their targets more directly with lower doses. This approach facilitates higher drug concentrations in the inner ear while minimizing systemic side effects. Currently, two primary routes for local drug delivery to the inner ear are intravestibular delivery (intracochlear) and intratympanic (extracochlear) application to the round window membrane. While direct inner ear opening for drug delivery is rare in humans, intratympanic drug delivery is widely used for various inner ear conditions such as idiopathic sudden sensorineural hearing loss and Meniere's disease (24). Among the various local delivery systems, nanoparticle-based delivery has emerged as promising, offering advantages in targeted drug delivery (25, 26).

The objective of this present study is to leverage the advantages of the multimodal porphyrin-based nanoparticles. First, an investigation of their safety was performed after intratympanic injection. Thereafter, their penetration ability and efficiency as drug delivery systems to the inner ear were studied. Lastly, the potential of porphyrin-based nanoparticles as a PTT agent for the treatment of cholesteatoma was investigated. Overall, we aimed to investigate their potential use in the middle and inner ear, while first establishing their safety for prospective applications.

## 2 Methods

### 2.1 Ethics approval

All experimental procedures on animals in this present study were approved by the Animal Care Committee of the Sunnybrook Research Institute, which adheres to the Policies and Guidelines of the Canadian Council on Animal Care and meets all the requirements of the Provincial Statute of Ontario Animals for Research Act together with those of the Canadian Federal Health of Animals Act.

### 2.2 Cochlear explant culture

#### 2.2.1 Preparation of Matrigel-coated glass-bottom culture dishes for cochlear explant

Culture dishes were coated using Matrigel as previously described with some modifications (27). A pre-aliquoted 150 µl of Matrigel (Corning Cat. No. 356234) was thawed on ice and mixed with 2.5 ml of Gibco Neurobasal-A Medium (1×). Subsequently, the mixture was vortexed and kept on ice for stability. Thereafter, approximately 120 µl of the Matrigel–Neurobasal mixture was added to the 10 mm well of each glass-bottom culture dish (MatTek P35G-0.170-14-C). The Matrigel works on improving the adherence of the explants and increasing their growth and survival rate. The culture dishes, along with a small dish of water to maintain humidity, were placed inside the incubator (27).

#### 2.2.2 Preparation of media

Culture media were prepared using a method described previously with some modifications (27). For each 10 ml volume, the proportions were 9.69 ml of Neurobasal-A Medium (1×, Gibco, 10888-022), 100 µl of N-2 supplement (100×, Gibco, 17502-048), 100 µl of L-glutamine (200 mM, Gibco, 25030-149), 1 µl of D-glucose (Gibco, 15023-021), 10 µl of ciprofloxacin (Sigma-Aldrich, 85721-33-1), and 100 µl of amphotericin B (250 µg/ml, Gibco, 15290-026) (27). Five milliliters of HEPES solution (1 M, Wisent Inc., 330-050-EI) were added to 500 ml of Hank's Balanced Salt Solution (HBSS) (1×, Wisent Inc., 311-513-CL). The mixture was dispensed into a 50 ml tube for the collection of neonatal mice heads post-decapitation and was put on ice (27). The dissecting media comprised 50 ml of 1% HEPES in HBSS. Amphotericin B (250 µg/ml, Gibco, 15290-026) was incorporated at a proportion of 1:100, and ciprofloxacin (Sigma-Aldrich,17850-5G-F) was introduced at a proportion of 1:1,000 to the dissecting media (27).

#### 2.2.3 Harvest of cochlear explants

As per the American Veterinary Medical Association (AVMA) guidelines, decapitation of pups (P0–P2) was quickly performed using sharp surgical scissors (28). The decapitation procedure was performed according to the animal care standardized practice. Dissection protocol of CD-1 pups’ inner ear was performed as described in detail by Meas et al. and Ogier et al. with some modifications. After decapitation, the heads were dropped into ice-cold HBSS with 1% HEPES on a dissecting plate to separate the organ of Corti. The bony cochlear wall was gently removed, then the spiral ligament/stria vascularis unwound from the modiolus, and the dissected cochleae were finally transferred to a Matrigel-coated plate, ensuring the correct orientation with the organ of Corti facing upward. After the inner ear explants underwent overnight incubation for optimal adhesion, the media were changed the following day (27, 29).

#### 2.2.4 Porphysome *ex vivo* cytotoxicity study

On the fourth day of explant culture, the culture media were aspirated from the plates. Subsequently, 100 μl of 0.1% Triton X-100 (Sigma-Aldrich, 9002-93-1), 100 μl of culture media, and 100 μl of the porphysome nanoparticles (ePS, nPS, and PS) at different concentrations (0.2, 2, 5, 50, 100 μM) were prepared in the explant culture media and added to the explants. Porphysomes were prepared and generously supplied by GZ's lab in Princess Margaret Cancer Centre (10, 14, 30). The plates were then returned to the incubator for a 30 min exposure period. Following treatment, the explants underwent a thorough washing procedure with media, involving three cycles of 5 min each. To assess the percentage viability of the explants, alamarBlue reagent was used.

#### 2.2.5 alamarBlue viability

alamarBlue measures quantitatively the reducing power of living cells (31). The assay and data analysis were performed as previously described by Longhin et al (32). Briefly, after the desired treatment and washing step, the media were aspirated from the wells of the explants and replaced with 80 µl of alamarBlue reagent diluted in culture media at a ratio of 1:10 followed by incubation at 37°C, 5% CO_2_ overnight. Thereafter, the alamarBlue was aspirated and transferred to a tissue culture-compatible 96-well plate. The fluorescence was measured using absorbance at 570 and 600 nm. The obtained viability results were compared across the different nanoparticle types and concentrations. Statistical analysis was performed using ordinary one-way ANOVA tests and Dunnett's multiple comparisons test. Data were expressed as mean ± SEM (*n* = 7–12).

#### 2.2.6 Immunohistochemistry of explants

The protocol used is as described by Ogier et al. with some modifications. Briefly, after treatment and thorough washing, explants are fixed with ice-cold 4% paraformaldehyde (PFA) and washed thrice with 1× phosphate buffered saline (PBS) (Wisent Inc., 811-012 FL). The explants are then incubated overnight at 4°C with 80 µl of 5% donkey serum (Sigma-Aldrich, D9663-10ML). The cells were then subjected to staining with primary antibodies Myosin VIIA (rabbit anti-Myosin VIIA from Proteus BioSciences, USA, at a 1:500 dilution) in 1% donkey serum overnight at 4°C. Subsequently, the cells underwent incubation with rabbit anti-Alexa 488 green (Thermo Fisher Scientific Inc., A21206) secondary antibodies at a 1:1,000 dilution for 1.5 h at room temperature protected from light. DAPI staining (Invitrogen, 00-4959-52) was performed during the mounting step. Imaging was conducted using the Nikon confocal microscope. The count of outer and inner hair cells was performed manually in the microscopic field using a 100 μm drawn long rectangle box. Hair cell count was performed across three different regions of each explant with three replicates (*n* = 3). The statistical analysis was performed using two-way ANOVA and Dunnett's multiple comparisons test (33).

### 2.3 *In vivo* cytotoxicity study

Adult male Long Evans rats weighing approximately 300 mg were obtained from Charles River Laboratories, ON, Canada, at an age of 5 weeks and acclimatized to the institutional vivarium for 1 week. Approximately 20 µl of different agents were injected into the middle ear cavity through the tympanic membrane. Subsequently, hearing, including distortion product otoacoustic emissions (DPOAEs) and auditory brainstem response (ABR), and vestibular/behavioral testing were performed at baseline and at 2 and 6 weeks postinjection (34–36). Finally, immunohistochemistry was performed after the sacrifice at 6 weeks’ time point.

#### 2.3.1 Intratympanic administration

Isoflurane 4 L/min (Fresenius Kabi AG, Homburg, Germany) was used to anesthetize the rats until rats were unresponsive to pain stimuli. The microscope (Wild Heerbrugg MTR29, Leica, Wetzlar, Germany) was then used to inspect the tympanic membrane to rule out pathologies. The intratympanic injection of 50 µM of different porphysomes (PS, ePS, nPS) and PBS as control was performed with a 25 G needle (BD, Franklin Lakes, NJ, USA) in the superior–posterior quadrant to ensure the most efficient administration to the round and oval window niche in the rat. The animal was then laid on the contralateral side with the injected ear facing up for 10 min to maximize inner ear absorption. Each group contained *n* = 6, and in every animal, the treated ear was randomized, and the other ear was injected with PBS as a control.

#### 2.3.2 Audiometric testing

Assessments were conducted before, as well as at 2 and 6 weeks following the initiation of intratympanic injection, as described above, of three different porphysome nanoparticles (ePS, PS, and nPS). This was performed using the Tucker-Davis Technologies (TDT) RZ6 system (34–36). Rats were anesthetized with isoflurane 4L/min followed by a 7 ml/kg saline solution containing 10% ketamine (100 mg/kg; Vetoquinol N.-A. Inc., Lavaltrie, QC, Canada) and 5% xylazine (20 mg/kg; Elanco Canada Limited, Guelph, ON, Canada). Briefly, as previously described, in a soundproof box, TDT acoustic system speakers were positioned directly into the ear canal for DPOAEs or 4 cm away from the ear during ABR conduction of acoustic stimuli. ABR tone stimuli at frequencies ranging from 4 to 32 kHz at intensities decreasing by 10 dB from 100 to 10 dB were delivered to the rats, and the lowest sound level was recorded, i.e., ABR threshold, at which a reproducible waveform was detected. Statistical analysis was performed using two-way ANOVA with Tukey's multiple comparisons test and *n* = 6–7 for all groups. Data collected represent the mean ± SEM and were used later to plot the graphs.

Regarding DPOAE assessments, OAEs were recorded by applying two continuous tones simultaneously (frequencies 1 and 2). Sound levels were then tested in increments of 10 dB starting from 80 to 20 dB. The distortion product was measured in response to f1 and f2. DPOAEs were measured at the center frequencies from 4 to 32 kHz. Thresholds of DPOAEs were recorded as the lowest noise level to observe a DP over background noise. Statistical significance was determined using two-way ANOVA with Tukey's multiple comparisons test and *n* = 6–7 for all groups. Data plotted represented the mean ± SEM.

#### 2.3.3 Vestibular and behavioral testing

Briefly, as described previously (34–36), the forced swim test was performed in a transparent water tank, 55 cm in length, 38 cm in width, and 25 cm in height, filled with room temperature water to 30 cm from the bottom of the tank. Each rat was placed at a time in the middle of the tank and left for a total of 6 min (2 min pretest and 4 min actual test). The mobility time was recorded with a stopwatch by two independent investigators. The time of mobility was then subtracted from the 240 s of the actual test time resulting in the total time of immobility. The procedure used was described by Can et al. (37). Statistical analysis was performed using ordinary one-way ANOVA tests and Tukey's multiple comparisons test. Data were calculated and plotted on graphs as mean ± SEM. The number of animals per group for each strain is 6–7.

As described previously, a beam test was conducted on the same day prior to the forced swim. It was performed using a wooden beam of 1 m long and 63 mm wide. It was placed horizontally without inclination and 45 cm away from a padded bottom container. Each rat was placed at a time on one side of the beam apparatus and encouraged to move by placing a peanut on the other end of the beam. On the test day, the time taken to cross each beam was recorded. Practice runs were performed before the actual test run; thereafter, two successful trials, where the rat did not stall, were averaged for analysis. Video recordings were used for detailed assessment of slipping and other motor deficits. The apparatus was cleaned between trials with towels soaked in 70% ethanol and water (38). Statistical analysis was performed using ordinary one-way ANOVA tests and Tukey's multiple comparisons test. Data are expressed as mean ± SEM, and the number of animals per group was 6–7.

#### 2.3.4 Cochlear whole-mount histological analysis

Upon completing 6 weeks post-intratympanic injection, rats were euthanized. The procedure was performed, according to animal care standardized practice, by inhalation of a lethal dose of isoflurane in oxygen as carrier (concentration of 5%) using a vaporizer in a closed chamber (39). Rats were further kept in the chamber for a minimum of 2 min after their lack of response to stimuli and respiratory depression to confirm cardiac arrest, and the heart was then promptly removed to ensure death (40). The cochleae were then harvested, and a whole-mount procedure was performed as previously described (34, 35, 41). Briefly, the round and oval windows were flushed using 4% paraformaldehyde, followed by overnight incubation in 2 ml of 4% paraformaldehyde at room temperature on a shaker. Subsequently, the cochlea underwent a 48 h incubation in 5 ml OsteoSoft (Merck KGaA) at room temperature on a shaker. Using a dissecting microscope, the organ of Corti was carefully dissected out in HBSS. The apex, mid-turn, and basal turn were segregated, and surrounding tissue was meticulously removed from the hair cells. The isolated hair cells were placed in petri dishes and incubated overnight at 4°C with 80 µl of 5% donkey serum. The cells were then subjected to staining with primary antibodies anti-Myosin VII A (Proteus BioSciences, USA, used at 1:500) diluted in 1% donkey serum overnight at 4 °C. Subsequently, the cells underwent incubation with rabbit anti-Alexa 488 green secondary antibodies at a 1:1,000 dilution for 1 h and 30 min at room temperature protected from light. DAPI staining (ProLong® Gold Antifade Reagent with DAPI, Life Technologies) was performed during whole-mount processing. Imaging was conducted using the Nikon confocal microscope with various objective digital zooms. The images were then captured with the same settings and were processed using software (Adobe Photoshop, ImageJ). The count of outer and inner hair cells was performed manually in the microscopic field using a 100 μm drawn long rectangle box. Various random sections along the cochlear duct were used and averaged for statistical analysis. Non-parametric two-way ANOVA test with Dunnett's multiple comparisons test was used to determine any statistical significance.

### 2.4 Quantitative analysis of *in vivo* porphysome penetration into the inner ear post-intratympanic injection

**Figure 1:**
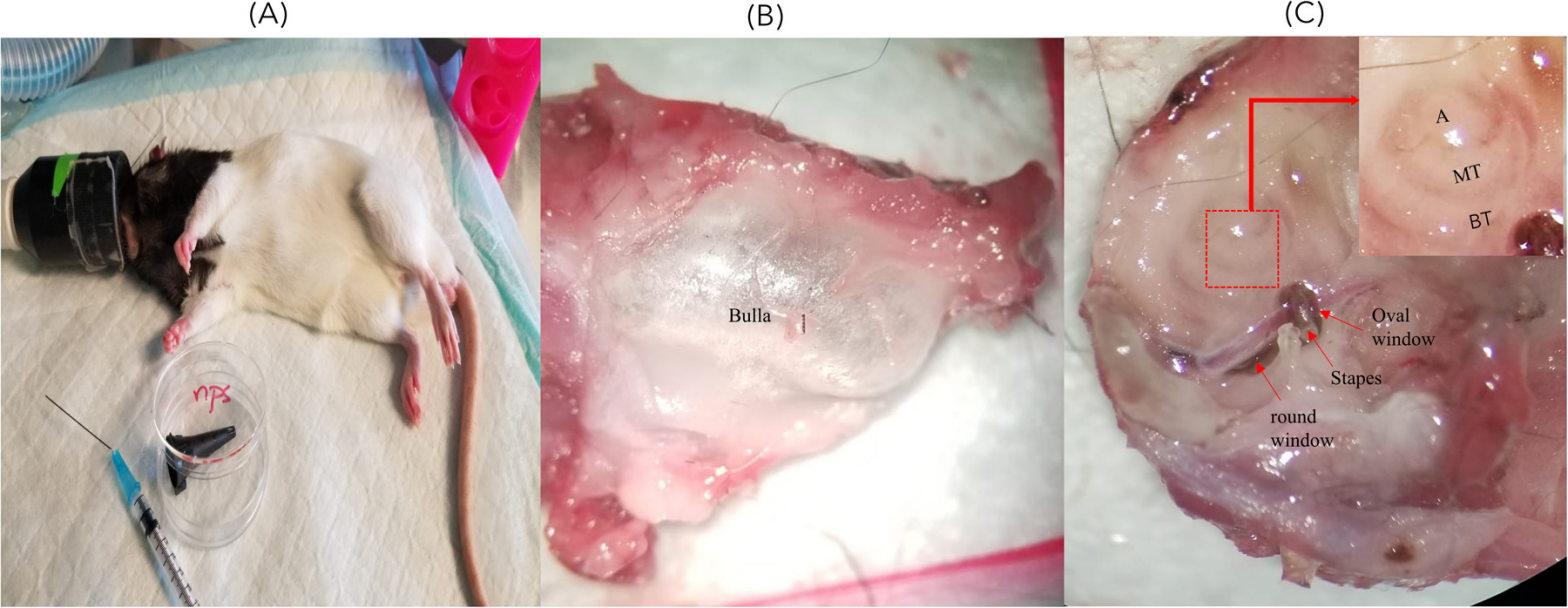
**(A)** Rat position during IT injection. This image shows the rat inclined to the dorsal side with the injected ear facing upward and slightly backward. **(B)** Whole-mount dissection of the cochlea showing the dissected cochlea before the removal of the bulla. **(C)** The dissected cochlea after the removal of the bulla showing the stapes and round and oval windows where the perilymph sample was collected. A, apex; MT, middle turn; BT, basal turn.

To examine penetration into the inner ear, intratympanic injection was performed as described above. Different increasing concentrations of ePS [50 (*n* = 4), 250 (*n* = 4), and 1,000 µM (*n* = 4), ∼50 μl] into the middle ear were injected through the tympanic membrane. Thereafter, rats were positioned with the injected ear tilted upward for 1 h as seen in Figure 1A. We included two control groups; one was intratympanically (IT) injected with PBS and sacrificed after 1 h (*n* = 6), while the other group was injected with 250 µM ePS and was sacrificed immediately after injection at (*t* = 0) (*n* = 4). Following 1 h, rats were sacrificed, and the perilymph and cochleae were collected to detect fluorescence signal via FluoroMax Plus Compact Steady State Spectrofluorometer and confocal microscopy. Regarding perilymph collection, an incision was made in the neck to open the skull. Thereafter, the brain was removed, and the temporal bone was excised from the skull base (Figures 1B,C). The bulla was then opened, and the cochlea was washed multiple times with PBS and dried with a clean tissue to ensure uncontaminated sampling. Subsequently, perilymph collection was performed from the round window after removing the stapes with a modified 25 G needle. Worth mentioning, the bevel of the needle had to be cut to reduce the lumen of the tip to fit through the round window. Thereafter, a 10 µl capillary micropipette (Drummond, PA, USA) was used to extract the microsample from the needle tip to the Eppendorf, put on ice, and transferred directly to the Princess Margaret facility to be analyzed via FluoroMax Plus Compact Steady State Spectrofluorometer to detect any fluorescence signal of the porphysomes. Statistical analysis was performed using ordinary one-way ANOVA tests and Tukey's multiple comparisons test. Data were expressed as mean ± SEM. The number of animals per group was 4–6.

### 2.5 *Ex vivo* cholesteatoma experiment

#### 2.5.1 Collection of cholesteatoma tissues and photothermal experiment on human cholesteatoma tissue

Cholesteatoma tissues were anonymously collected from study subjects (*n* = 6). The study was approved by the Research Ethics Board at Sunnybrook Research Institute, REB #1662. Initially, ePS, nPS, and PS were prepared at a concentration of 1 mM, followed by dilution to achieve a final concentration of 50 μM using PBS. Cholesteatoma tissues of each subject were cut into four equal pieces and added to a 1 ml aliquot of the 50 μM porphysome suspension. Cholesteatoma tissue in DMEM/F12 without porphysomes served as a negative control. The samples were incubated for 20 min under conditions simulating the middle ear, specifically 5% CO_2_ and 37°C. Following incubation, each tissue sample was placed in a petri dish, positioned 1 cm away from a 671 nm laser with a fluence rate of 750 mW/cm^2^, for irradiation. The samples were exposed to light, and concurrent recordings of temperature using a thermal camera were executed as described previously in detail (42). The irradiation period spanned approximately 5 min, after which the samples were transferred to 2 ml Eppendorf tubes containing 4% PFA for fixation and subsequent preparation for histological examination (11). Statistical analysis was performed using ordinary one-way ANOVA tests and Dunnett's multiple comparisons test. Data are expressed as mean ± SEM. The number of cholesteatoma tissues per group is 6.

#### 2.5.2 Primary keratinocyte isolation and cell culture

A previously published protocol was used to isolate and characterize the cholesteatoma keratinocytes (43). Concisely, cholesteatoma tissues were surgically resected from study subjects and promptly transported to our laboratory on ice. On the same day of the collection, the tissues were then carefully diced and put in 2 ml Eppendorf of 200 U/ml collagenase IV in DMEM/F12 (Cat. No. LS004188, Worthington Biochemical Corporation, 730 Vassar Ave, NJ 08701, USA) overnight at 4 ℃, employing a shaker to facilitate tissue dissociation. Following the overnight treatment, 3 ml of 10% FBS in DMEM/F12 was added to the digested tissues to deactivate the collagenase activity. The digested tissues were pipetted up and down 100 times. Subsequently, the mixture underwent filtration using a 4 µM filter. The resulting filtrate was subjected to centrifugation at 1,500 rpm for 5 min at room temperature. After discarding the supernatant, the pellet was inoculated with KSFM medium (Cat. No. 17005042, Invitrogen, Carlsbad, CA, USA) enriched with 500 U/ml streptomycin/penicillin (Cat. No. 15070063, Invitrogen, Carlsbad, CA, USA) in a 25 cm^2^ cell culture flask. The cultures were maintained under 5% CO_2_ and 37 ℃ conditions. KSFM media and antibiotics were changed every 3 days (44). The cell cultures from the second passages were utilized in the following assays, maintaining a cell culture density of 1 × 105 cells/cm^2^ for all assays unless specified otherwise. Passaging of keratinocytes was performed as described (45). The required volume of 0.05% trypsin–EDTA solution was heated to 37 °C in an incubator. Subsequently, the medium of the keratinocyte cell culture was aspirated, and 1 ml of the pre-warmed 0.05% trypsin–EDTA solution was added to the cells. The flask was placed back in the incubator (37 °C), and after 5 min, microscopic inspection was conducted to determine if the cells had initiated detachment. Once roughly 50% of the cells had loosened, the culture flask was gently tapped against the hand to facilitate the detachment of the remaining cells. The cell suspension was then transferred to a 50 ml tube. Subsequently, 3 ml of DMEM/F12 + 10% FBS (37 °C) was added to the cell suspension to deactivate the trypsin. The culture plate was rinsed with 1 ml of DMEM/F12 + 10% FBS, and the rinse was added to the 50 ml tube. The cells were centrifuged for 10 min at 450 × *g* at room temperature. The pelleted cells were resuspended in 1 ml of KSFM (37 °C). A cell count was conducted, and 200,000 cells were transferred to a 25 cm^2^ culture flask, along with 5 ml of KSFM. The culture flask was gently agitated to ensure an even distribution of the cells and put back in the incubator.

##### 2.5.2.1 Cytotoxicity study on keratinocyte using ePS without photothermal laser

For this experiment, keratinocyte cells were cultured as described above in 196 microplate wells at a density of 12,000 cells and with 90 µl of the KSFM per well. Cell culture was left for 3 days to grow in the incubator. On the fourth day, cells were incubated with PBS (control) and 5, 10, and 50 µM ePS for 30 min and 24 h (*n* = 3 per group). Thereafter, cells were washed with PBS, and 10 µl of alamarBlue was added to 90 µl of the cell suspension and incubated at 37°C for 1–4 h. Absorbance signal was read using an absorbance plate reader, and concentration was then calculated. Data were analyzed using two-way ANOVA and Dunnett's multiple comparisons test. The experiment was repeated three times, and the average and SEM were calculated and plotted.

## 3 Results

### 3.1 Investigation of cytotoxicity of porphysomes on cochlear explants

**Figure 2:**
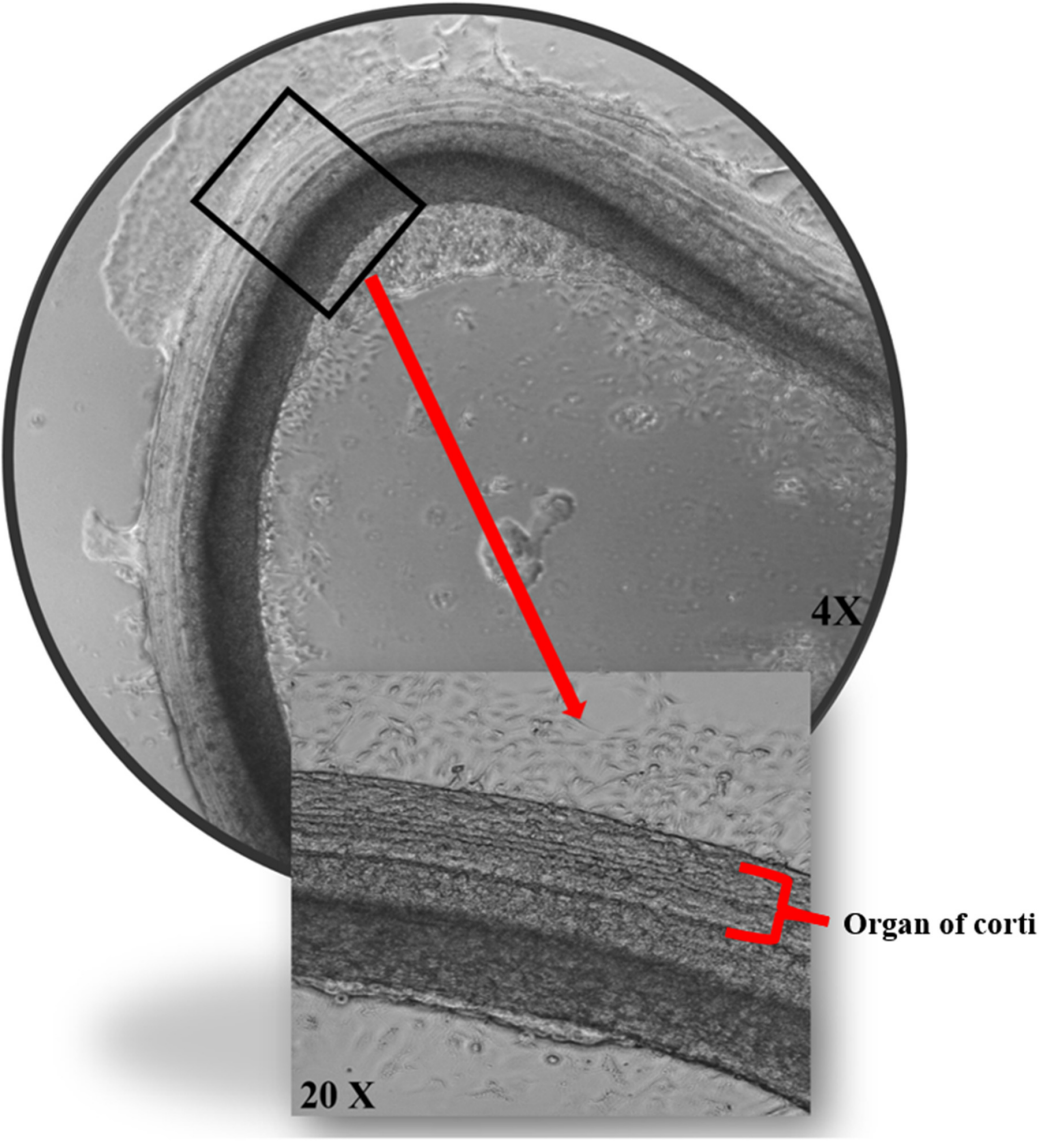
The effects of porphysomes on cochlear explants. Cochlear explants from our work under transmitted light microscopy posttreatment with ePS showing intact three outer rows and one inner row of hair cells.

**Figure 3:**
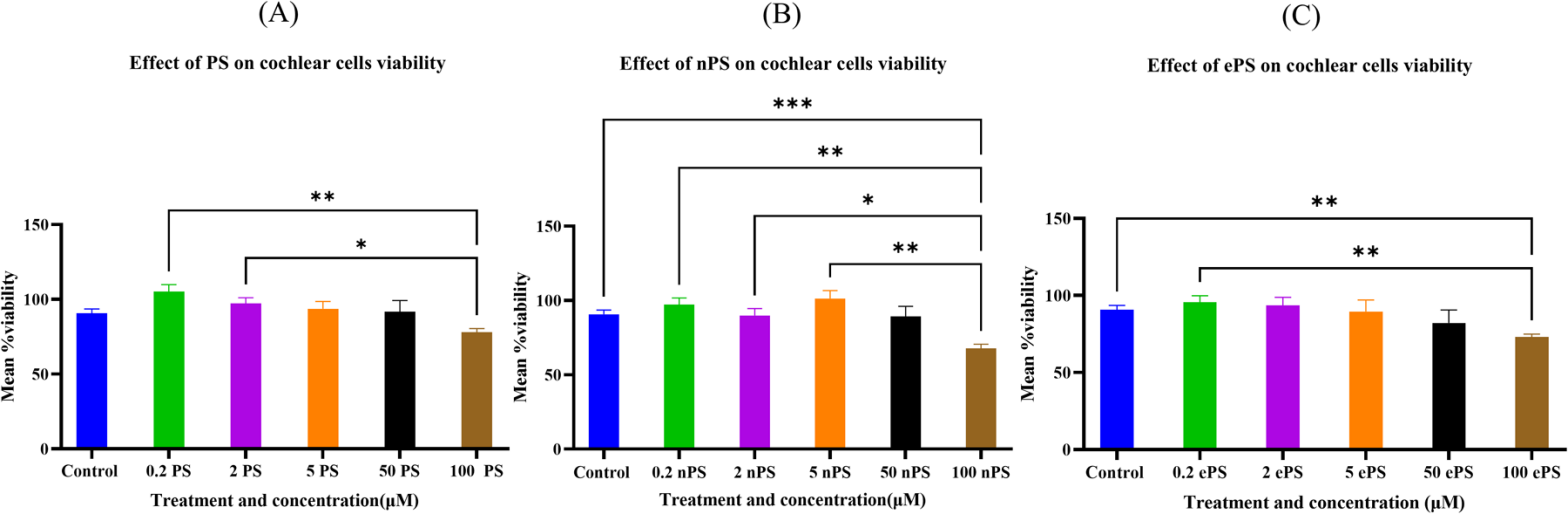
Cytotoxicity assessment of cochlear explants post porphysome treatment. The cell activity determined by the alamarBlue assay. Experiments were performed under six different conditions: no treatment (PBS treated), 0.1% Triton, and 0.2, 2, 5, and 50 μM and 100 mM of ePS, nPS, and PS (*n* = 7–12). All comparisons were statistically significant. Data are expressed as mean ± SEM, where **p < 0.0085 and ***p < 0.0009. **(A)** Mean % viability of cochlear explants post PS exposure, **(B)** mean % viability of cochlear explants post nPS exposure, and **(C)** mean % viability of cochlear explants post ePS exposure.

The alamarBlue assay was used to assess the cell activity of cochlear explants dissected (Figure 2). Data showed, as demonstrated in Figure 3, no significant difference up to 50 µM of concentration across all porphysome types when compared with the control (*p* > 0.2614). In contrast, the 0.1% Triton control group showed a statistically significant difference when compared with all the conditions. Moreover, 100 µM concentration showed a significant difference when comparing ePS and nPS to the control (*p* < 0.0016). PS showed no significant difference up to 100 µM concentration (*p* = 0.07).

**Figure 4:**
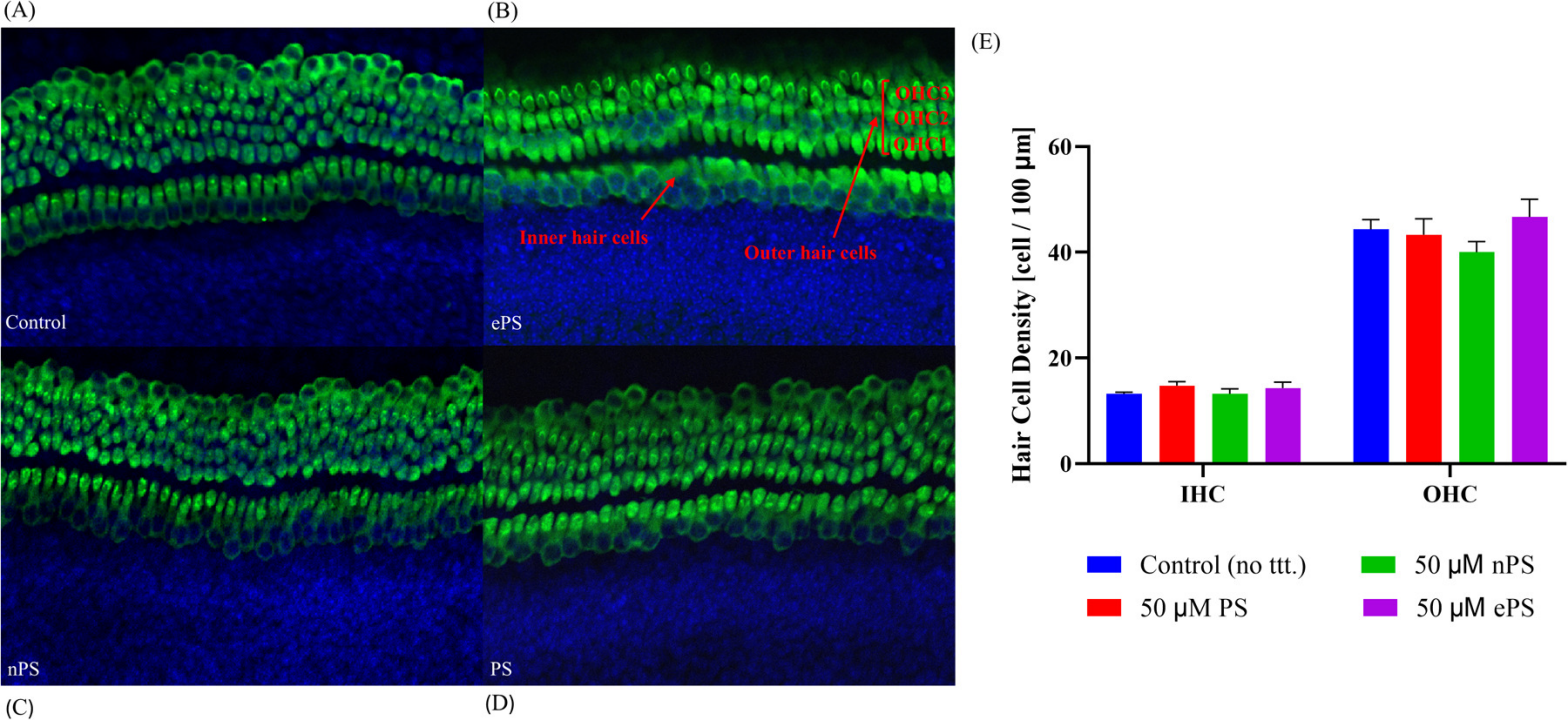
The effects of porphysomes on cochlear hair cells. The mechanosensitive hair bundles (HB) of hair cells are seen on top of each hair cell **(A–D)**. They are clusters of stereocilia and can be stained with Myosin VIIA. The hair bundles labeled Myosin VII after 30 min of porphysome treatment. In the control group **(A)**, the three rows of outer hair cells and one row of inner hair cells retained the original regular arrangement and V-shaped hair bundles. Similarly, explants treated with 50 µM ePS **(B)**, 50 µM nPS **(C)**, or 50 µM PS **(D)** showed preserved hair cell arrangement together with the hair bundles and no appearance of missing hair cells. **(E)** Hair cell count of cochlear explants post porphysome exposure. The graph represents the number of Myosin VII-positive IHCs or OHCs per 100 µm calculated from apex, middle, and basal turn of cochlear explants, expressed as a mean ± SEM (*n* = 3 different explants per group); *p* > 0.6220, compared with untreated control (PBS exposed). HB, hair bundles; IHC, inner hair cells; OHC, outer hair cells.

In addition to assessing cytotoxicity, immunohistochemistry was performed where we focused on the organ of Corti region to evaluate the effects of porphysomes (46). Similarly to the untreated controls which showed a striped pattern with translucent bands at the organ of Corti (Figure 4), cochlear explants treated with ePS, nPS, and PS revealed the same appearance (Figures 4B–D). In the control cochlear explants, three rows of outer hair cells and one row of inner hair cells retained the original regular arrangement and the V-shaped hair bundles (Figure 4A). Similarly, the porphysome-exposed cochlear explants showed a similar hair cell arrangement with the appearance of hair bundle, and no missing hair cells were observed.

Subsequently, hair cell count of inner and outer hair cells was performed to detect any difference or missing hair cells in porphysome-exposed explants when compared with control-treated explants as seen in Figure 4E. Hair cell count was performed across three different regions of each explant across a 100 µm cross-diameter with three replicates (*n* = 3). The data showed no statistical significance between any of the porphysome-exposed explants when compared with the control explant exposed to phosphate buffer saline.

### 3.2 ABR amplitude data at 2 and 6 weeks after porphysome intratympanic injection suggest no hearing loss

**Figure 5:**
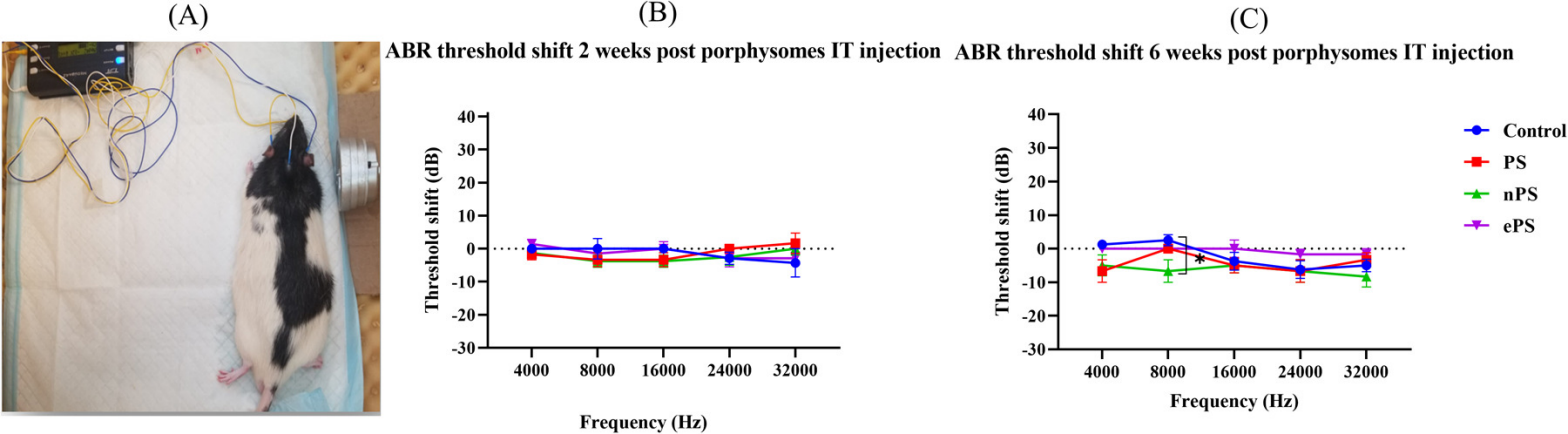
ABR threshold shift at 2 and 6 weeks postinjection. **(A)** Auditory brain stem response test demonstration. **(B)** ABR threshold shift 2 weeks post porphysome injection. **(C)** ABR threshold shift 6 weeks post porphysome injection. ABR was measured at baseline and 2 and 6 weeks post PBS and porphysome injection. Hearing tests were performed at 4, 8, 16, 24, and 32 kHz. Threshold change was calculated by subtracting the pretreatment threshold from the posttreatment threshold. The threshold was graphed on the *y*-axis in dB. Wave I amplitude measured in nanovolts using the MATLAB program was plotted on the *y*-axis. Intensities starting at 90 dB and decreasing by 10 dB decrements were graphed on the *x*-axis for each frequency. PBS was graphed in blue, PS in red, nPS in green, and ePS in purple. Data plotted here represent the mean ± SEM, where *p < 0.02; otherwise, data were not significant.

Functional testing, ABR, and DPOAEs were performed at 2 and 6 weeks posttreatment. At 2 weeks’ time point post porphysome injection, there was no significant difference between the threshold shift at any of the frequencies when comparing the PS-, ePS-, and nPS-injected groups with the control group (PBS injected) as demonstrated in Figure 5B. Similarly, there was no significant difference seen across all the frequencies after 6 weeks post porphysome injection when comparing the PS- and ePS-injected groups with the control group. However, there was a significant difference (*p* < 0.0266) detected when comparing the nPS-treated and control groups where the threshold shift was lower for the nPS-injected group as seen in Figure 5C.

### 3.3 Shift at 2 and 6 weeks after porphysome intratympanic injection suggests no hearing DPOAE threshold loss

**Figure 6:**
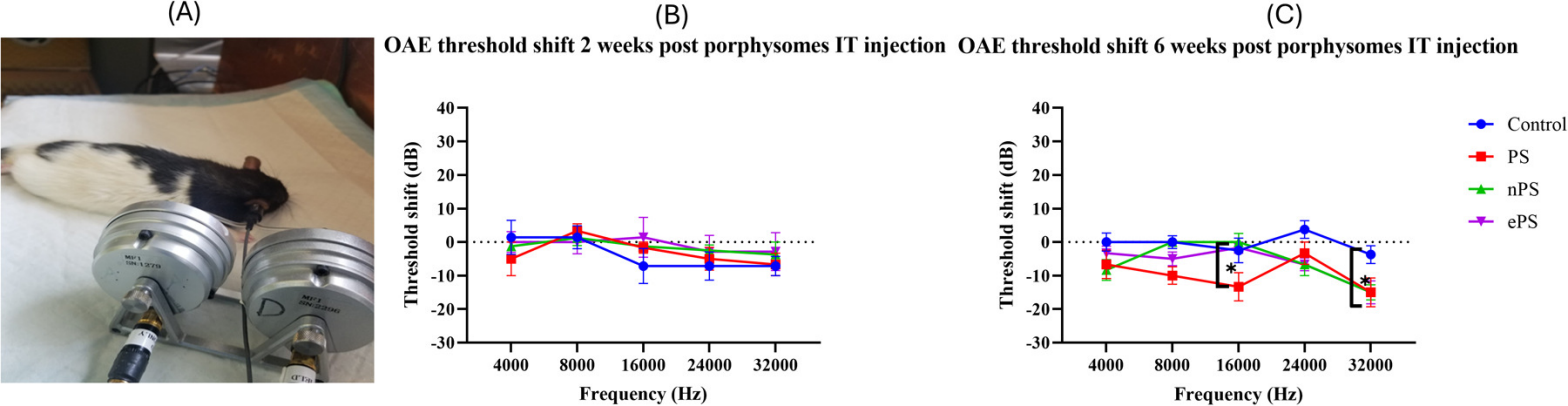
DPOAE threshold shift at 2 and 6 weeks postinjection. **(A)** Demonstration of OAE experiment and rat position. **(B)** DPOAE threshold shift at 2 weeks postinjection. **(C)** DPOAE threshold shift at 6 weeks postinjection. DPOAE was measured at baseline, 2 and 6 weeks post saline and porphysome injection. Hearing tests were performed at 4, 8, 16, 24, and 32 kHz. Threshold shift was calculated by subtracting the pretreatment threshold from the posttreatment threshold. The threshold was graphed on the *y*-axis in dB. Intensities starting at 100 dB and decreasing with 20 dB decrements were graphed on the *x*-axis for each frequency. Saline was graphed in blue, PS in red, nPS in green, and ePS in purple. Plotted here is the mean ± SEM, where *p < 0.038; otherwise, data was not significant, *p* > 0.05.

DPOAE was performed at baseline and 2 and 6 weeks post PS, ePS, and nPS injection to the middle ear. Demonstration of the OAE experiment and rat position during tests can be seen in Figure 6A. At 2 weeks’ time point post porphysome injection, there was no significant difference between the threshold shift at any of the frequencies when comparing the PS-, ePS-, nPS-injected groups and the control group (PBS injected) as demonstrated in Figure 6B. At 6 weeks’ time point (Figure 6C), there was a significant difference detected at 16 kHz between the PS-injected group compared with all the other three groups (control, nPS, and ePS). At 32,000 kHz, there was also a significant difference detected between the PBS group and the PS-, ePS-, and nPS-injected groups. However, all measurements at both frequencies were below 0 dB threshold shift.

### 3.4 Vestibular findings 6 weeks post porphysome intratympanic injection

**Figure 7:**
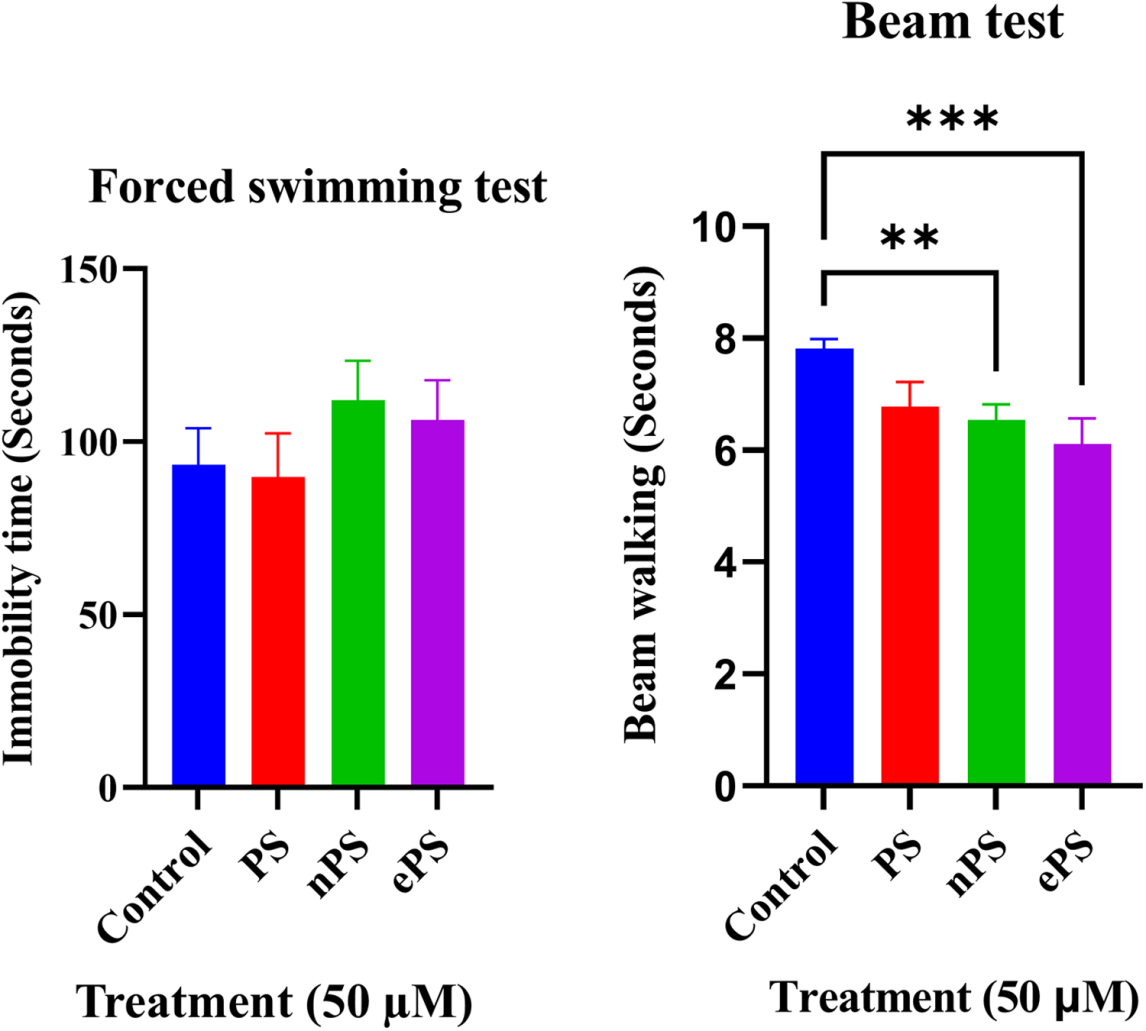
Forced swim and beam tests 6 weeks post porphysome IT injection. **(A)** Compared with the control, there was no significant difference when comparing the immobility time with any of the porphysome-injected groups (*p* > 0.7237). **(B)** Compared with the control, there was no significant difference with PS-injected groups (*p* = 0.0617). However, there was a statistical significance difference between the control and nPS, *p* = 0.0091, and ePS, *p* = 0.0008.

Vestibular function was evaluated through the swim test and beam test performed after 6 weeks of intratympanic injection of 50 µM of the different porphysomes (three different exposed groups) and PBS as control, as seen in Figure 7A. Compared with the control group, immobility time of the ePS, nPS, and PS groups showed no statistically significant differences (*p* > 0.6651).

The beam test was used to assess motor and balance coordination. The time taken for the rats to cross the beam without slipping was documented three times, and the obtained values were averaged. Data in Figure 7B represent the results 6 weeks postinjection compared with the control group. Data showed a significant difference between the control, ePS, and nPS where the time taken by the nPS- and ePS-injected groups was significantly shorter than the control group (*p* < 0.05). Specifically, there was a statistically significant difference between the control and nPS (*p* = 0.0091) and ePS (*p* = 0.0008). However, there was no significant difference between the control and PS-injected group (*p* = 0.0617).

### 3.5 Integrity of outer and inner hair cells post porphysome intratympanic injection at the apex, middle, and basal turn

**Figure 8:**
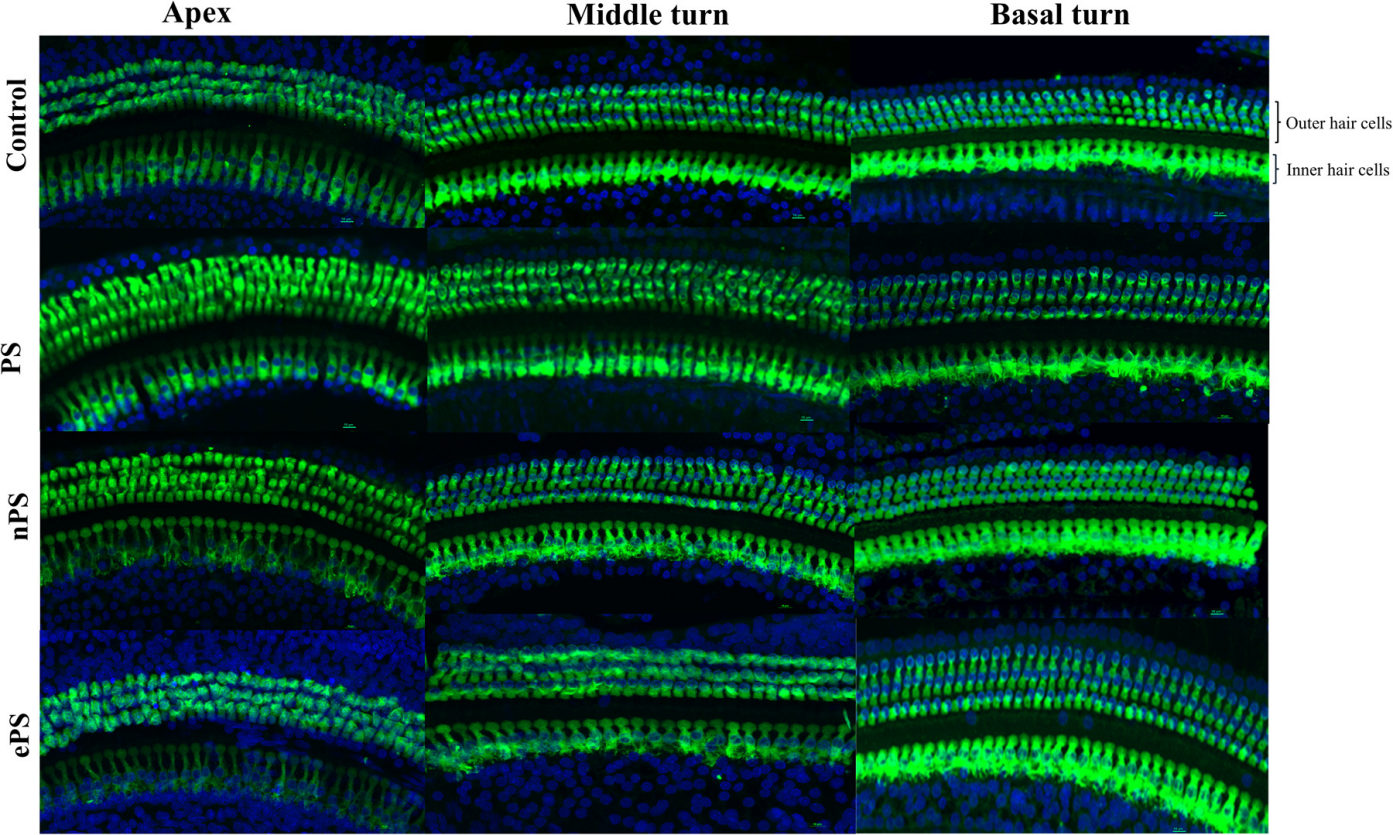
Whole-mount post porphysome intratympanic injection. At the 6-week time point post porphysome injection, rats were euthanized, and their organ of Corti epithelial layer was dissected out and stained with Myosin VIIA for outer hair cell staining. Images were taken using a Nikon confocal microscope, and count was performed across 100 µm sections with three replicates across the three turns. This panel represents the apex, middle, and basal turn showing IHCs and OHCs of the different treatments at 20× objective. Multiple sections were analyzed from each turn taken from *n* = 7 ears for the treated groups and *n* = 7 for the PBS group. Results showed no statistically significant difference between all groups, where *p* > 0.6162 for basal hair cell count, *p* > 0.4968 for middle turn, and *p* > 0.7403 for apex.

At the end of the protocol, rats were euthanized, and cochleae were collected for histological testing. Inner and outer hair cell morphology was labeled with Myosin VIIA in response to different treatments at different cochlear regions shown in Figure 8. Intact hair cell morphology was seen, without difference between the groups intratympanically injected with porphysomes and the control group, in the apical, middle, and basal regions (Figure 8). The mean inner hair cell count for all groups was 10 ± 1 per 100 µm cross section, while the outer hair cell count (OHC) was 37 ± 1 per 100 µm cross section. There were no missing inner or outer hair cells or any empty spaces seen across all turns.

### 3.6 *In vivo* ePS penetration to the inner ear post-intratympanic injection

**Figure 9:**
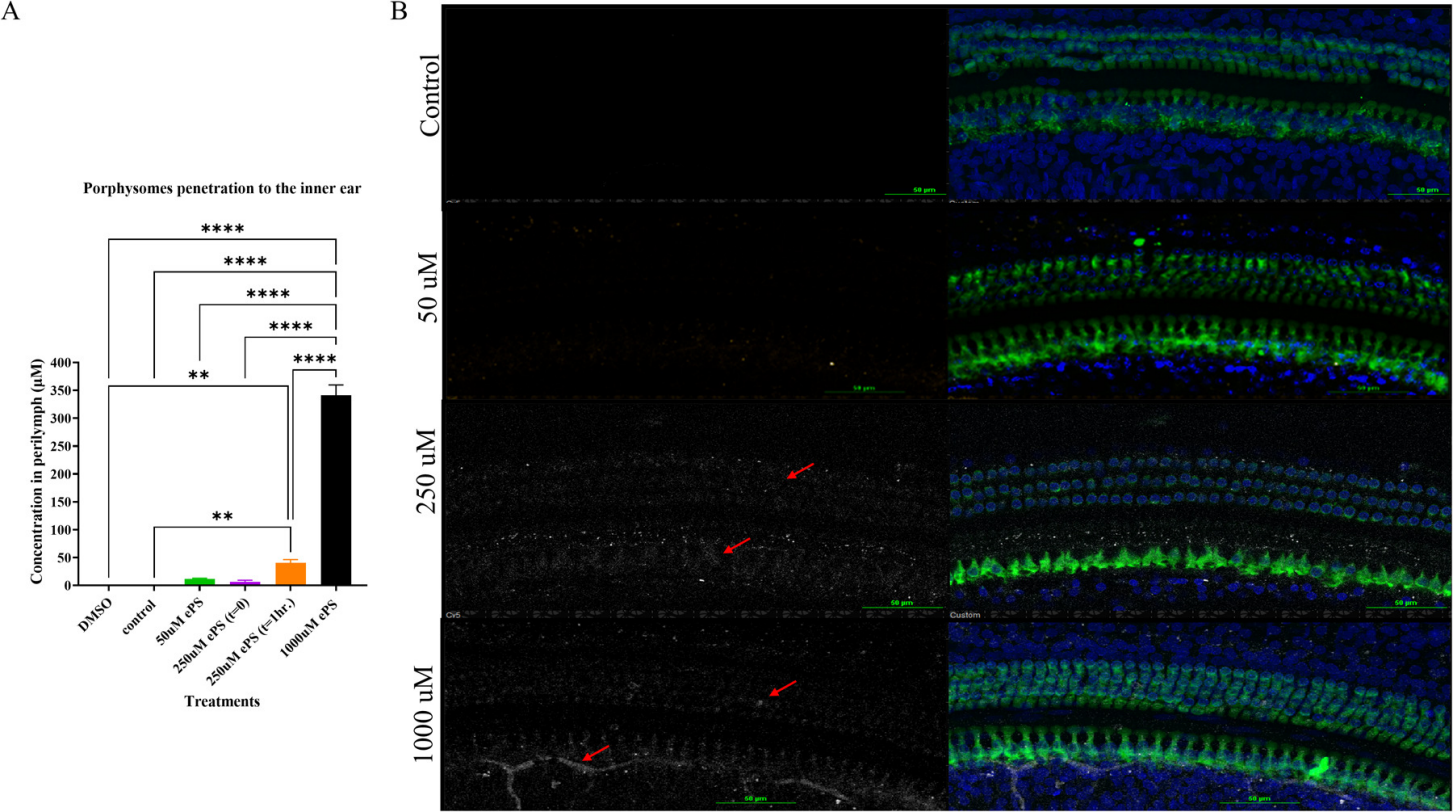
**(A)** Concentration of ePS nanoparticles in the perilymph. Data are expressed as mean ± SEM, **p < 0.0054, ****p < 0.0001. **(B)** Confocal microscopy image of the cochlea at the middle turn after 1 h of the ePS exposure at concentrations of 50, 250, and 1,000 µM and at *t* = 0 for 250 µM ePS. The slices were immunolabeled with Myosin VIIA (Myo7A) for stained hair cells, and ePS signal was detected in the Cy5 channel, white signal (red arrows), (*n* = 3).

We were able to detect a fluorescence peak signal at 675 nm in the perilymph samples collected from all the ePS-exposed groups as seen in Figure 9. This peak is characteristic and specific to the porphysomes. Compared with the control, there was only a significant difference between the 1,000 and 250 µM at *t* = 1 h, and results were otherwise insignificant. Data in Figure 9A showed that for the 1,000 µM that was originally injected, approximately 341.0902 µM ± 18.61642 was detected in the perilymph at *t* = 1 h (*p* ≤ 0.0001). Out of the 250 µM, approximately 40.2122 µM ± 6.187139 was detected in the perilymph at *t* = 1 h (*p* = 0.0054). Out of the 250 µM, 6.568226 µM ± 2.764485 was detected at *t* = 0 (*p* = 0.9913). Out of the 50 µM, 11.9338 µM ± 0.983321 was detected in the perilymph at *t* = 1 h (*p* = 0.8395). The control showed no peak at 657 nm.

Immunohistochemistry and confocal imaging allowed for the visualization of the organ of Corti, and we were able to detect the fluorescence of ePS in the treated inner ears but none in the control ear, as shown in Figure 9B. The fluorescence was unspecific and seen around the nuclei of the inner and outer hair cells as well as supporting cells. In 1,000-µM-treated groups, porphyrin signal seems to be detected in nerves as well.

### 3.7 *Ex vivo* cholesteatoma photothermal therapy

**Figure 10:**
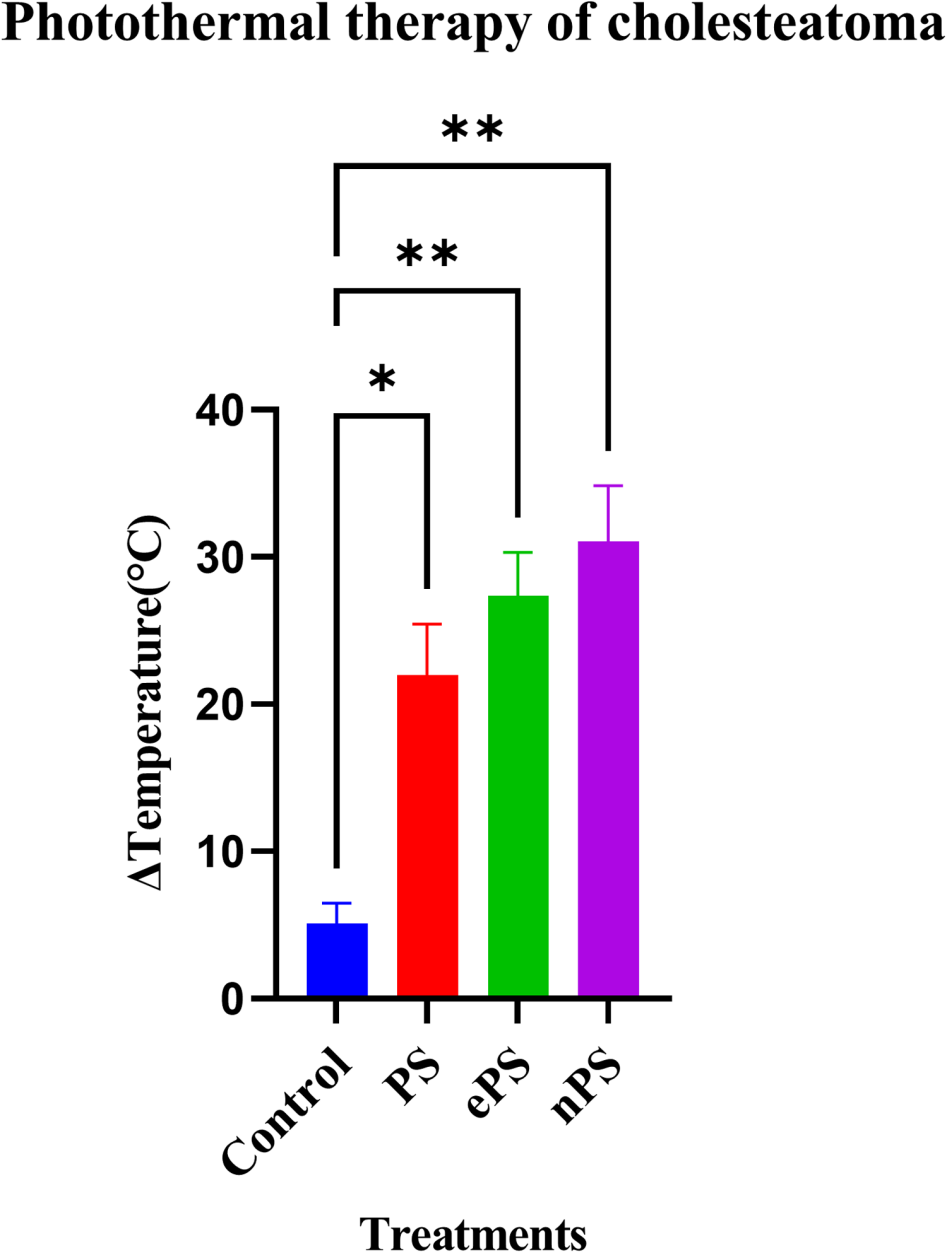
Photothermal therapy effect of porphysomes on cholesteatoma tissue. Concentration of ePS, PS, and nPS is 50 µM in all treatments. Data on the *x*-axis represents the difference in temperature before laser irradiation and the maximum temperature that was reached while irradiation. *p = 0.0199 and **p < 0.0032.

**Figure 11:**
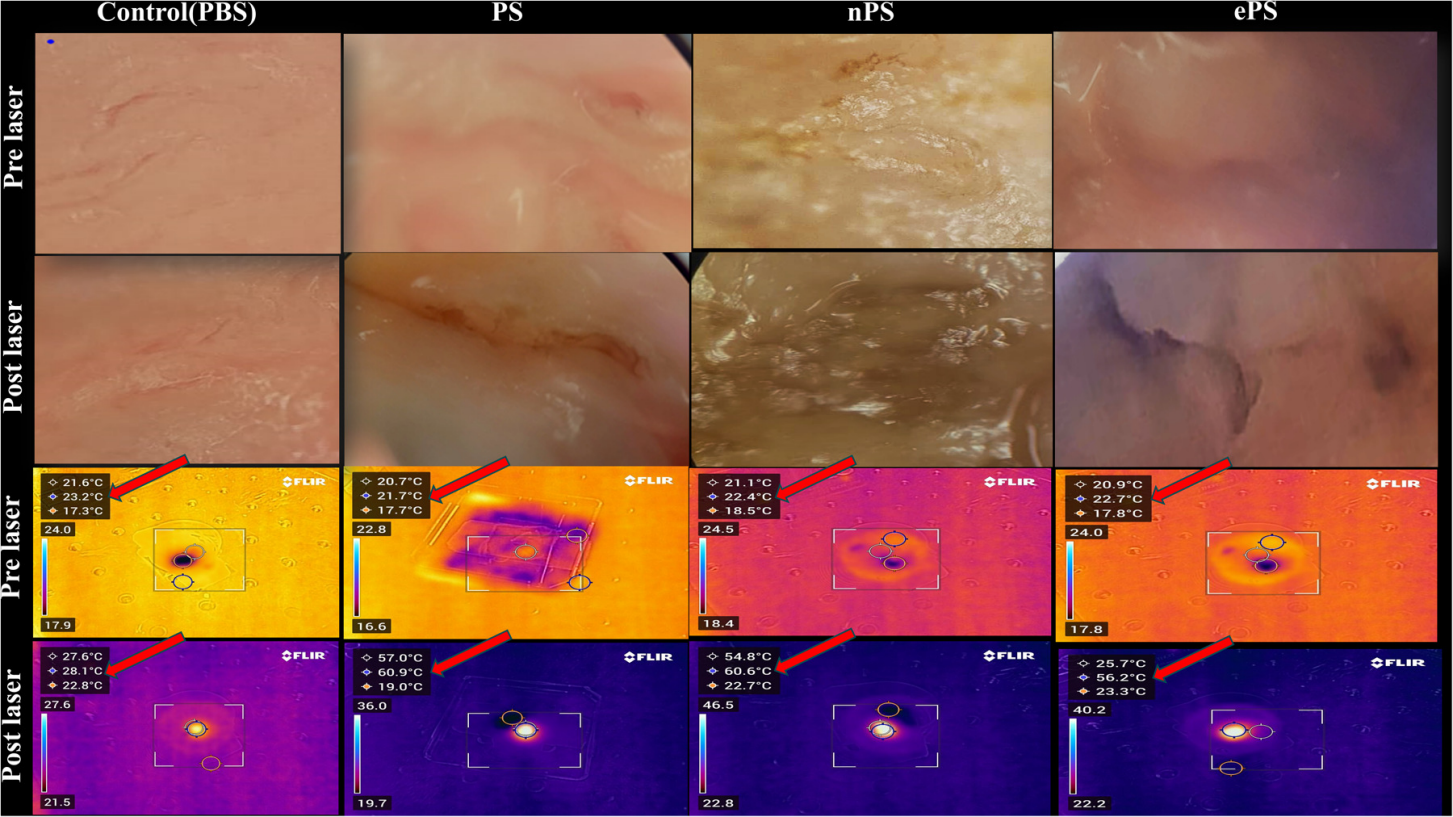
Cholesteatoma photothermal therapy microscopic and thermal camera images. The vertical columns represent the different treatments while the horizontal rows represent the microscopic and thermal camera images pre- and post-laser for each corresponding treatment. The temperature scale bar illustrates the temperature change. The highest temperature reached is the middle temperature (red arrow).

**Figure 12:**
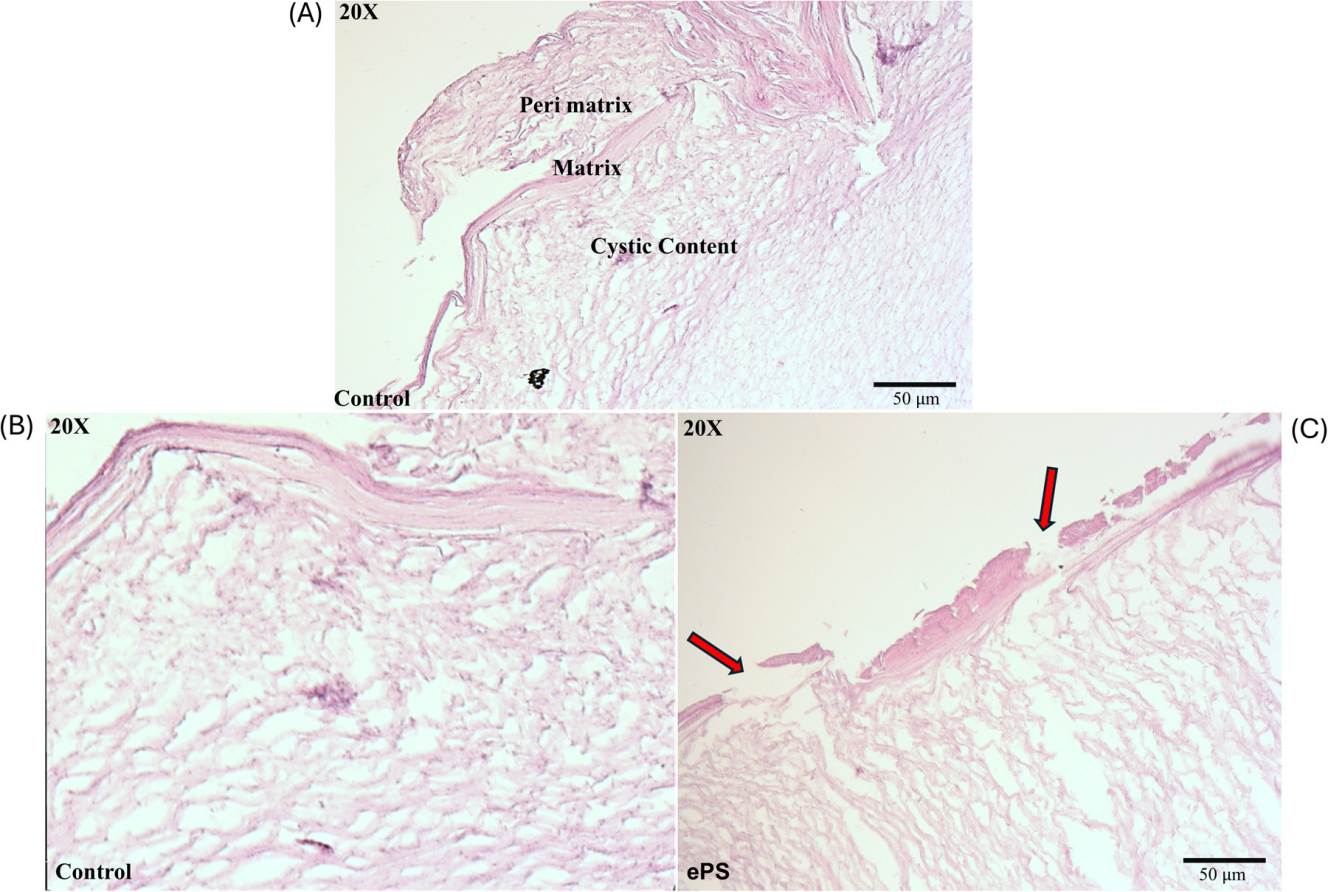
Histology of cholesteatoma pre- and post-laser photothermal therapy. Images of cholesteatoma specimens are taken using H&E staining at 20× magnification. **(A)** Image of cholesteatoma dissected in our lab showing the different layers: perimatrix, matrix, and cystic content similar to a reference of cholesteatoma histology image (47). **(B)** Cholesteatoma tissue post (laser + PBS) exposure. **(C)** Cholesteatoma tissue post (laser + 50 µM ePS) exposure showing damage or breaks in perimatrix and matrix (red arrows).

As demonstrated in Figure 10, we were able to monitor the temperature increase of cholesteatoma tissues during NIR laser irradiation *in vitro* using a stand-held thermal camera. Surface temperature of cholesteatoma was monitored to gauge the distribution of heat for PS, nPS, and ePS compared with the control exposed cholesteatoma (PBS exposed). Results in Figure 10 show a significant difference when comparing the temperature of all (laser + porphysomes) exposed cholesteatoma samples when compared with (laser + PBS) exposed cholesteatoma samples. The temperature difference is calculated by subtracting the pre-laser temperature from the post-laser temperature. Data showed that nPS resulted in the highest temperature increase (Δ*T* = 31.07 ± 3.76, *p* < 0.0001). ePS followed nPS in increasing the temperature of cholesteatoma (Δ*T* = 27.38 ± 2.93, *p* = 0.0001), while PS showed a temperature increase of Δ*T* = 21.98 ± 3.48362 (*p* = 0.0023). Furthermore, the temperature increase observed by infrared thermography was corroborated by microscopic images and histology, as seen in Figures 11 and 12. It is demonstrated that higher temperatures (caused by laser + porphysomes) match pronounced burn marks on cholesteatoma tissues as seen with ePS and nPS compared with PS posttreatment samples or control samples.

The main histological changes are presented in Figure 12. Cholesteatoma is histologically composed of a cystic content (keratin lamellae), matrix (hyperproliferative stratified squamous epithelium), and a perimatrix (inflamed subepithelial connective tissue–granulation tissue) (47) as seen in Figure 12A. Compared with the control (PBS + laser) as in Figure 12B, cholesteatoma samples treated with laser + ePS, nPS, or PS showed obvious damage to the structure of the outermost layer composing the cholesteatoma. In Figure 12C, we demonstrate the effect of ePS compared with the control which shows obvious damage to the perimatrix and matrix (red arrows). ePS combined with laser treatment resulted in damage and pores to the matrix and perimatrix layers of the cholesteatoma unlike the treated cholesteatoma samples that were treated with PBS and laser which showed integrated cholesteatoma layers with no damage.

**Figure 13:**
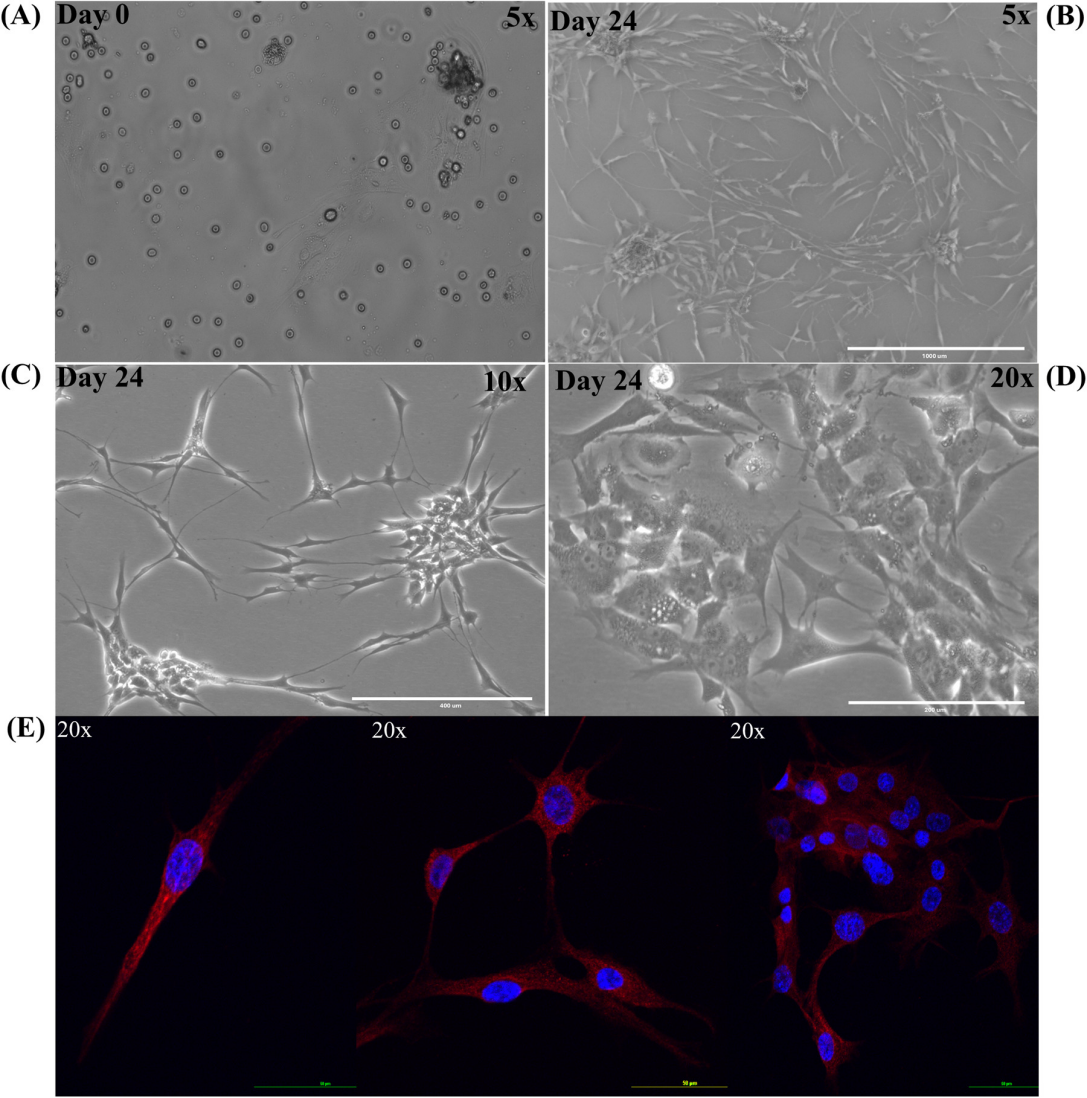
Keratinocyte primary cell culture from human cholesteatoma samples. Primary cell culture of keratinocytes. **(A)** Keratinocyte primary cell culture at day 0. **(B)** Keratinocyte primary cell culture at day 24 under 5× objective. **(C)** Keratinocyte primary cell culture at day 24 under 10× objective. **(D)** Keratinocyte primary cell culture at day 24 under 20× objective. **(E)** Keratinocytes were fixed, labeled with cytokeratin 16 (red) with nucleus (DAPI stain in blue).

Cholesteatoma, like skin, sheds keratin debris and stains positive for cytokeratin as seen in Figure 13 (48–50). In this *in vitro* study, we used the primary cell culture to test the effect of porphysomes on cholesteatoma, in an attempt to reach a concentration that is safe for surrounding normal tissue of the middle ear while being selectively toxic upon specific laser irradiation of the cholesteatoma tissue. Figures 13A–D present keratinocyte primary cell culture at day 0 and day 24 after culture from human cholesteatoma samples collected after surgery at various objectives. In addition, keratinocytes were fixed, labeled with cytokeratin 16, and imaged using confocal laser microscopy as seen in Figure 13E to confirm the keratinocyte cell culture.

**Figure 14:**
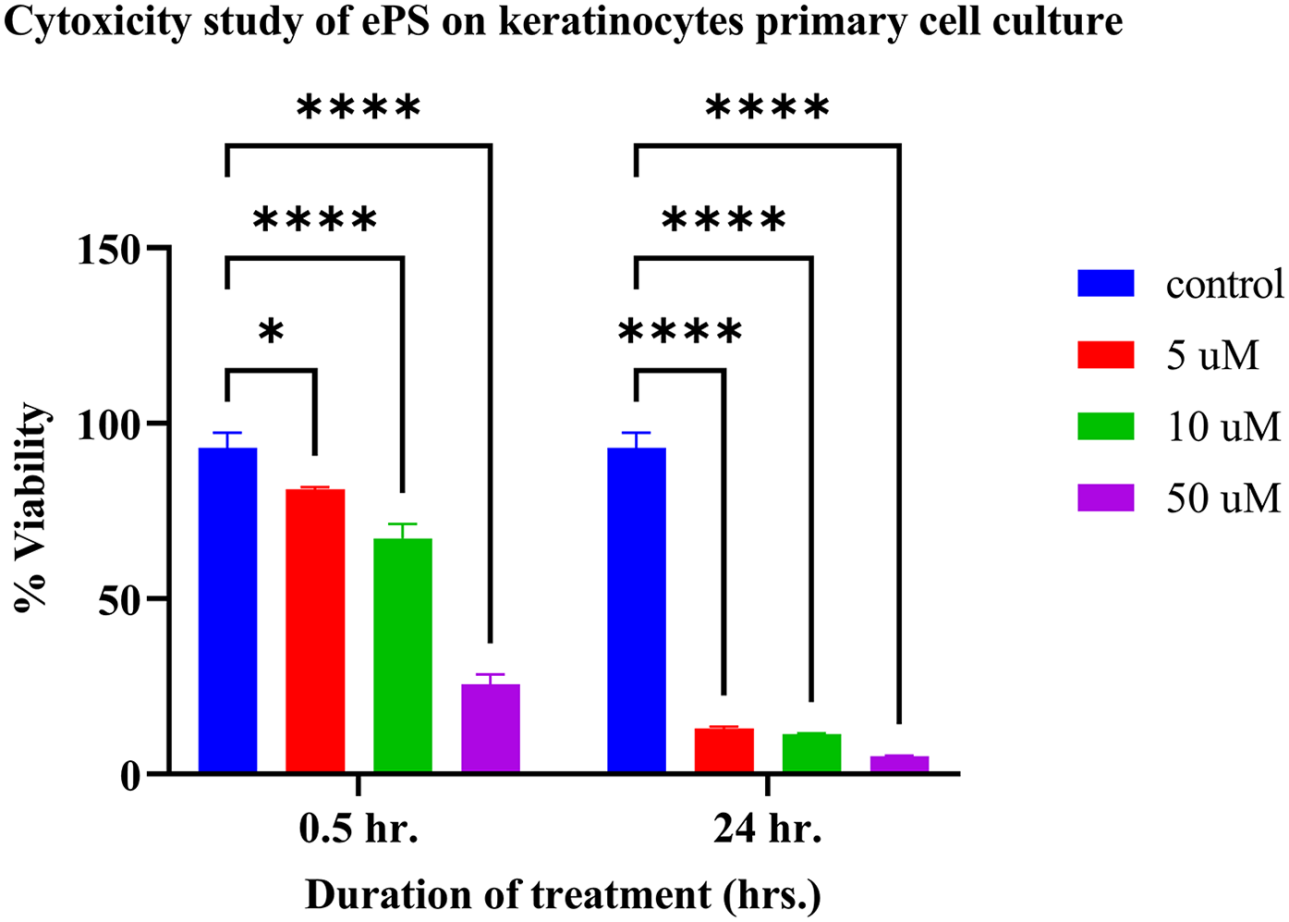
Toxicity study of ePS on keratinocyte primary cell culture plotting mean % viability of keratinocyte at 0.5 and 24 h. post ePS exposure. Results showed a significant difference with all treatments compared with the control (*p* < 0.05). *p = 0.0204, ****p < 0.0001.

The cytotoxicity experiment performed on the keratinocyte primary cell culture showed a significant difference between all treatments (*p* < 0.05), compared with the control with PBS (Figure 14). Regarding the 30 min incubation time with ePS, the mean % viability showed a dose-dependent effect; the lesser the viability, the higher the concentration (control: 93.00 ± 4.28%; 5 µM: 81.13% ± 0.64%; 10 µM: 67.19% ± 4.08%; 50 µM: 25.57% ± 2.83%) (*n* = 3). Regarding the 24-h contact time with ePS, the mean % viability of control and 5, 10, and 50 µM concentrations was 13.01% ± 0.61%, 11.47% ± 0.17%, and 5.25% ± 0.05%, respectively, (*n* = 3).

## 4 Discussion

This study is the first to investigate the application of this multimodal porphyrin-based nanoparticles in the inner ear and explore the highest and safest concentration of the three different novel porphyrin-based nanoparticles, i.e., PS, nPS, and ePS. Other studies have investigated the toxicity of nanoparticles based on the particle size in other tissues, specifically on HEI-OC1 cochlear cells and on HaCaT keratinocytes (51). In our study, the viability of the cochlear hair cells after 30 min exposure to the nanoparticles was assessed by using the alamarBlue fluorometric test as previously performed (51). The observed variation, as shown in Figure 3, in safety thresholds among the three porphyrin-based nanoparticles likely stems from differences in their colloidal stability and cellular uptake. These results could be attributed to the better colloidal stability and, hence, better uptake of the nPS by the hair cells, resulting in higher toxicity at increased concentration of nPS compared with PS (14). Regarding ePS, the inclusion of aminopolycarboxylic acid-conjugated lipids, such as EDTA- or DTPA-hexadecylamine lipids in liposome-like porphyrin nanoparticles (PS) resulted in enhanced intracellular uptake by 25-fold, owing to the lipids’ ability to fluidize the cell membrane in a detergent-like manner instead of metal chelation of EDTA or DTPA which similarly can cause toxicities at higher concentration if compared with the prototype liposome-like porphysomes, i.e., PS (30).

After penetration into the inner ear, porphysomes seem to distribute along the entire cochlea through the perilymph and endolymph fluids. In our study, we detected a concentration-dependent penetration to the inner ear, suggested by the increasing intensity of the specific fluorescence peak at 675 nm detected (Figure 9A) (52). Histology showed a strong fluorescent signal of ePS in all the inner ear cell types in a concentration-dependent manner, with the highest signal being for the concentration of 1,000 µM ePS followed by the 250 µM ePS, as shown in Figure 9B. Interestingly, there was no significant difference between the 50 µM concentration and control. Going forward, this is a crucial finding for planning future studies targeting nanoparticles for inner ear drug delivery, with 250 µM being the concentration of choice. This choice selection is supported by *in vivo* data, where an initial predelivery concentration of 250 µM results in a final intracochlear concentration of approximately 50 µM after 1 h of exposure (Figure 9A). Notably, this postdelivery concentration remains within the safety threshold established *in situ* (Figure 3C), where 50 µM showed no significant toxic effects compared with control. These findings reinforce the suitability of 250 µM as an optimal starting concentration for nanoparticle-based inner ear drug delivery, ensuring both effective drug exposure and biocompatibility.

To further confirm that ePS nanoparticles penetrate the inner ear and that the signal detected in the perilymph is not due to cross-contamination, we injected intratympanically 250 µM and PBS in the experimental group and control group, respectively. Subsequently, each subject was sacrificed immediately after injection. However, it is worth mentioning that it takes approximately 12 min for the sacrifice to be performed, and some ePS can readily penetrate the inner ear effectively. This justifies the signal detected in the perilymph samples of the 250 µM group at (*t* = 0). However, the signal was insignificant when compared with the control group. This experiment suggests that penetration is valid and not due to contamination from the middle ear during sample collection.

Confocal images, as seen in Figure 9, showed that ePS manifested strong fluorescence around the inner hair cells and spiral ganglion neurons. Some fluorescence was also seen around the nucleus of the outer hair cells. This unspecific uptake resulted from the ability of ePS to fluidize the cell membrane and penetrate intracellularly due to the incorporation of aminopolycarboxylic acid-conjugated lipids, such as EDTA- or DTPA-hexadecylamine lipids in porphyrin nanoparticles (PS) which potentiated their uptake intracellularly by 25-fold (30).

The influence of EDTA was recently investigated when cells were cultured with different concentrations of EDTA-lipid (ePS_med_ and ePS_high_) to explore the influence of nanoparticles containing ≥30 mol% of EDTA-lipid compared with PS (without EDTA) (30). The study confirmed that the highest uptake was the nanoparticles containing the highest concentration of EDTA. Due to that specific reason, we chose ePS to be tested in our *in vivo* study, being a promising candidate for inner ear drug delivery. To achieve specific cell targeting in the cochlea, conjugation of known ligands to surface receptors of selected cells needs to be conjugated to engineered NPs. For example, ePS surface functionalized with a nerve growth factor-derived ligand (hNgf EE peptide) represents a promising potential for targeting specifically to spiral ganglion neurons (52).

To investigate the safety of porphysomes functionally and histologically, we performed hearing tests, balance tests, and confocal laser microscopy of the organ of Corti (53, 54). The ABR and OAE results of the functional tests (Figures 5, 6) showed no significant difference between any of the baseline hearing measurements and 2 weeks postinjection measurements among all porphysomes at all frequencies (*p* > 0.05). At 6 weeks’ time point, the result is the same except for a significant difference between the nPS-treated group and the control group at 8,000 Hz, showing that the nPS-injected group had a lower hearing threshold, i.e., better hearing. However, the difference is just above 5 dB, and it is likely attributed to different days of measurement and rater technical measurement variation. We also argue that measurements on different days could result in such variations due to a slightly different layout of the experiment. Impaired hearing function is reflected in the behavioral findings in the swim test and beam test (55). While normal rats are expected to swim and float without any problems, rats with vestibular impairment sink or have difficulties in swimming and floating (56). Increased time or slips while finishing the course of the beam are related to motor impairment due to hearing impairment (55, 57). During the swim testing in our study, there was no significant increase in the immobility time as well as motor coordination in the beam test when comparing the control group with groups 6 weeks post porphysome injection (Figure 7). The only statistically significant difference was detected when comparing the control group with nPS and ePS 6 weeks postinjection. This statistical significance suggests that the post porphysome-injected group was faster in finishing the course of the beam, and this could be attributed to the fact that the rats, 6 weeks postinjection, were more trained to finish the beam course and took less time to traverse the beam, which further suggests the safety of the porphysomes for inner ear drug delivery.

On the histological level (Figure 8), safety was demonstrated by the integrity of the organ of Corti cell structures. A thorough cell count was performed across the apex, middle, and basal turn to detect any differences in the histology or toxicity when comparing treated groups with the control groups. Toxicity would be manifested as damaged or missing hair cells (58, 59). Results showed no significant difference in the cell count between the porphysome-treated groups and the control, further suggesting safety. This study is crucial as effectively delivering therapeutic agents to the inner ear without damaging the integrity of delicate structures is one of the greatest challenges (52). These results align with the safety data of previous research using porphysomes intravenously (42).

As seen in Figure 9A, in the case of middle ear application for phototherapy cholesteatoma treatment, 50 µM is deemed to be the chosen concentration, since it does not seem to diffuse as easily into the inner ear. In our study, we detected only an insignificant amount in the perilymph after intratympanic injection compared with the control, hence suggesting the safety of the inner ear structure when applying phototherapy. However, one of the limitations of this study is the absence of an *in vivo* model for cholesteatoma that evaluates the safety of the inner ear after PTT *in vivo*.

Since *in vivo* tumors were not included in our study, monitoring surface temperature was reasonable and served as an approximation of the effect of porphysomes on the whole cholesteatoma tissue (60). Although at the beginning of the experiment, PS showed a high increase in temperature, the mean average of the increase in temperature of nPS and ePS was higher at the end of the study (Figure 10). This could be attributed to the condition of the tissues used which was difficult to control in our study due to several factors. However, compared with the control, the thermal heat generated from all the porphyrin-based nanoparticles was significantly higher (*p* < 0.0199), suggesting the efficacy of PTT on cholesteatoma tissues. The phototherapy of these nanoparticles was extensively investigated in previous studies (30). In one study, ePS and PS showed obvious scabbing in KB tumor-bearing mice compared with the control, with ePS being superior. In another study, a large temperature increase (*T* > 52°C) was detected upon laser irradiation of nPS suggesting its efficiency in PTT, and a significant tumor growth inhibition was observed in mice treated with nPS (14).

In our study, the histological finding (Figure 12) showed a significant burn mark and damage demonstrated by the presence of breaks/pores and the detachment of the outermost layers of the cholesteatoma, the perimatrix and matrix. These layers are important for blood supply to the cholesteatoma to maintain its survival and growth (61). This has also been demonstrated in another study that investigated burn injuries on human skin explants showing the epidermis detached from the dermis (62). However, deeper tissue layers were not affected, and the burn was limited to the surface of the cholesteatoma as illustrated in our microscopic images as well (Figure 12C). The deeper layer contains debris that is the byproduct of dead skin cells. For comparison, control samples that were treated with PBS and laser only were included in our study which did not show any damage to any of the tissue layers, underscoring the thermal effect produced by the porphysomes in combination with the laser. However, the cholesteatoma *ex vivo* study bears the following limitations: lack of quantitative analysis of histological changes, lack of detection of ultrastructural changes of mitochondria and the organelles, and lack of expression levels of radical oxygen species and nitric oxide. This was dictated by the differences in tissue quality collected from patients and the transportation conditions between facilities. Overall, the integrity of the tissue samples was not standardized, and hence the results might not be consistent and must be interpreted with caution.

Subsequently, we performed an *in vitro* study on cholesteatoma keratinocyte primary cell culture. This study was performed to overcome the unstandardized conditions upon cholesteatoma tissue collection from the patients and to test for the effect of porphysome alone in a more consistent tissue live cell culture. The unlimited multiplication of keratinocytes is the key factor responsible for the pathogenesis of cholesteatoma in the middle ear. The objective of this study was to reach the concentration of porphysomes that maintains the viability of single-layer keratinocytes vs. causes cytotoxicity without laser application. ePS nanoparticles were selected in this study because they demonstrated to irradiate the highest temperature upon laser exposure in the previous *ex vivo* cholesteatoma experiment. The concentration and duration of exposure of 5 µM for 30 min exposure with the keratinocyte showed the least cytotoxic effect, with a cell viability of 81.13% ± 0.64% compared with the control. On the other hand, higher concentrations of ePS or longer exposure can lead to cytotoxicity of keratinocyte culture. Our findings indicate that ePS at low concentrations over extended exposure durations could serve as an effective standalone therapy to prevent keratinocyte survival in the middle ear, eliminating the need for laser photothermal therapy. Importantly, previous *in situ* cochlear explant studies suggest that 50 µM is a safe concentration for cochlear hair cells upon direct exposure for 30 min, with no observed ototoxic effects. Additionally, *in vivo* studies have shown that when 50 µM ePS is injected into the middle ear, no detectable nanoparticle penetration occurs within the first hour, further reinforcing its safety profile. The increased toxicity of nanoparticles toward rapidly dividing primary keratinocytes, compared with normal cells, can be attributed to the enhanced cellular uptake of nanoparticles by the former which exhibit higher endocytosis activity to support their increased metabolic demands. This heightened internalization makes them more susceptible to nanoparticle-induced damage (63, 64). These findings suggest that localized middle ear application of ePS at 50 µM could effectively target residual cholesteatoma cells while minimizing the risk of inner ear toxicity, making it a promising therapeutic approach for preventing recurrence after surgery. This result signifies the possibility of using ePS at 50 µM for multiple therapy sessions or at a low concentration but long duration as a single therapy to prevent keratinocyte survival in the middle ear without the need for laser photothermal therapy. This potentially can be used for small residual or recurrent keratin accumulation after middle ear surgery without the risk of inner ear penetration or ototoxicity.

## 5 Limitations

While our *ex vivo* findings demonstrate that 50 µM of porphyrin-based nanoparticles is safe for up to 30 min of contact with cochlear explants, several limitations should be noted. First, the ototoxicity evaluation was restricted to short-term, single-exposure conditions and did not assess long-term effects or repeated dosing, which are clinically relevant scenarios. Second, although we based our 30 min exposure window on prior literature, nanoparticle clearance and retention dynamics were not directly measured. Third, although 250 µM was identified as the optimal predelivery concentration based on perilymph drug levels, its safety was only demonstrated *ex vivo*; *in vivo* functional and histological assessments at this concentration remain necessary. Additionally, while we assessed hair cell viability, potential EDTA-related effects on tight junctions and other non-sensory cochlear structures were not specifically investigated. Despite the absence of functional impairment in hearing or balance, a more comprehensive histological evaluation, including ultrastructural and molecular endpoints, is needed to confirm long-term biocompatibility. Lastly, the absence of a validated *in vivo* cholesteatoma model limits our ability to fully assess the safety and therapeutic efficacy of photothermal treatment in a clinically relevant disease context.

## 6 Future direction

Future studies will aim to address these limitations through comprehensive *in vivo* evaluations of porphyrin-based nanoparticles. This includes long-term and repeated-dose safety studies, as well as pharmacokinetic analyses to quantify nanoparticle clearance, retention, and biodistribution. Functional and histological testing at the proposed 250 µM concentration is planned to confirm its safety and therapeutic viability *in vivo*. Mechanistic studies incorporating ultrastructural imaging and molecular markers of cellular stress will further clarify nanoparticle–tissue interactions. To establish clinical relevance, the development or implementation of a validated *in vivo* cholesteatoma model will be prioritized, enabling rigorous testing of photothermal therapy efficacy. Collectively, these investigations will help define the optimal therapeutic window and advance the clinical translation of porphysome-based therapies for inner and middle ear disorders.

## Data Availability

The raw data supporting the conclusions of this article will be made available by the authors, without undue reservation.
